# Liver Toxicity of Current Antiretroviral Regimens in HIV-Infected Patients with Chronic Viral Hepatitis in a Real-Life Setting: The HEPAVIR SEG-HEP Cohort

**DOI:** 10.1371/journal.pone.0148104

**Published:** 2016-02-05

**Authors:** Karin Neukam, José A. Mira, Antonio Collado, Antonio Rivero-Juárez, Patricia Monje-Agudo, Josefa Ruiz-Morales, María José Ríos, Dolores Merino, Francisco Téllez, Inés Pérez-Camacho, María Carmen Gálvez-Contreras, Antonio Rivero, Juan A. Pineda, HEPAVIR SEG-HEP-2007 Study Group of the Sociedad Andaluza de Enfermedades Infecciosas (SAEI)

**Affiliations:** 1 Unit of Infectious Diseases and Microbiology, Hospital Universitario de Valme, Seville, Spain; 2 Instituto de Biomedicina de Sevilla (IBiS), Seville, Spain; 3 Internal Medicine Service, Hospital Universitario de Valme, Seville, Spain; 4 Internal Medicine Department, Hospital Torrecárdenas, Almeria, Spain; 5 Unit of Infectious Diseases, Hospital Universitario Reina Sofia, Maimonides Institute for Biomedical Research (IMIBIC), University of Cordoba, Cordoba, Spain; 6 Unit of Infectious Diseases, Hospital Universitario Virgen de la Victoria, Malaga, Spain; 7 Unit of Infectious Diseases, Hospital Universitario Virgen de la Macarena, Seville, Spain; 8 Internal Medicine Service, Hospital Juan Ramón Jiménez. Huelva, Spain; 9 Unit of Infectious Diseases, Hospital de La Línea de la Concepción, Cadiz, Spain; 10 Unit of Tropical Medicine, Hospital Poniente, El Ejido, Spain; University of Pittsburgh Center for Vaccine Research, UNITED STATES

## Abstract

**Objective:**

To assess the current frequency of ART-associated grade 3–4 transaminase elevations (TE) and grade 4 total bilirubin elevations (TBE) in HIV-infected patients with chronic hepatitis B and/or C, who start a new regimen of ART.

**Patients and Methods:**

A total of 192 pre-treated or treatment-naive HIV infected patients with HBV and/or HCV-coinfection who started ART in eight Southern Spanish centers from July/2011-December/2013, were followed for 12 months in this prospective study.

**Results:**

Forty-one (21.4%) subjects had been naïve to ART, median (IQR) follow-up was 11.6 (5.6–12.9) months. The most frequently initiated NRTI were tenofovir/emtricitabine [49 patients (25.5%)]. Eighty-nine (46.4%) patients started a ritonavir-boosted protease inhibitor and 77 (40.1%) individuals a NNRTI. Raltegravir and maraviroc were initiated in 24 (12.5%) and 9 (4.7%) individuals. Ten [5.21%; 95% confidence interval (CI): 2.53%-9.37%] patients presented grade 3 TE, while 8 (4.17%; 95%CI: 1.82%-8.04%) subjects showed grade 4 TBE. No episodes of grade 4 TE or ART discontinuation due to hepatotoxic events were observed. The use of ritonavir-boosted atazanavir was the only independent predictor for grade 4 TBE [adjusted odds ratio: 7.327 (95%CI: 1.417–37.89); p = 0.018] in an analysis adjusted for age, sex and baseline HIV-RNA levels, while no factor could be independently associated with grade 3–4 TE.

**Conclusions:**

Currently, the frequency of severe ART-associated TE and TBE under real-life conditions in patients with chronic viral hepatitis is similar to what has been reported previously. However, episodes of grade 4 TE are less frequent and severe TE appears to be of lesser concern.

## Introduction

Hepatotoxic events associated with antiretroviral drugs, including liver transaminase elevations (TE), acute liver failure and death, are of high concern in HIV-infected patients. Coinfection with hepatitis viruses can promote the development of ART-induced TE [1–6]. Nevertheless, the number of patients with HCV and/or HBV coinfection included in clinical trials on various HIV drugs that assess TE is usually low [7–12].

Data on the current rates of TE observed among HIV-infected patients with hepatitis virus coinfection obtained in the clinical practice have been described within different cohorts [1, 3–5, 13–20]. However, most of these studies have a retrospective design. Additionally, often only specific ART regimens are considered, mainly ritonavir-boosted PI (PI/r) or the NNRTI efavirenz and nevirapine [1,4,5,13,14,16,20]. Also, real-life data on some drugs, such as rilpivirine, darunavir and dolutegravir in patients with HCV-coinfection are scarce, while other studies include concomitant antiretroviral drugs that are no longer recommended. Furthermore, in some of these studies, selection criteria lead to the exclusion of subjects according to their previous regimen or liver damage, or require an optimized background ART combination. Finally, the initiation of a NRTI within an existing regimen may play a role in the development of hepatotoxic events in this setting. Therefore, due to the regimens and study populations analyzed, the studies available to date may not represent the overall situation of rapidly changing ART strategies in the clinical practice. To date, there is little information on ART-induced hepatotoxicity based on regimens and changes reflecting real-life conditions. In this context, we reported an overall frequency of grade 3–4 TE of 7.6% for a HIV/HCV-coinfected population in a multicenter cohort study conducted from 2007 to 2009 [20]. Still, no changes in NRTI strategies were evaluated and liver toxicity assessment was limited to efavirenz and PI/r in combination with two fixed NRTIs. Taken together, this information is necessary to evaluate potential liver safety differences among currently used ART. These data might be used to optimize the management of HIV-infected patients with viral hepatitis.

The aim of this study was to evaluate the frequency of hepatotoxicity, determined by grade 3 or 4 TE and grade 4 total bilirubin elevations (TBE), in patients with chronic hepatitis B or C, during the first year after starting one or more currently used antiretroviral drug within one single cohort under real-life conditions.

## Patients and Methods

### Study Design and Population

Pre-treated or treatment-naïve HIV-infected patients who attended the Infectious Diseases Units of eight Southern Spanish centers from July 2011 to December 2013 were consecutively included in this prospective multicentric study (ClinicalTrials.gov ID: NCT01908660) if they fulfilled the following criteria: i) older than 18 years; ii) initiation of one or more antiretroviral drugs; iii) chronic HCV infection as confirmed by HCV antibodies in plasma, as well as a positive HCV viral load determined by PCR and/or chronic HBV infection as detected by positive serum HBV surface antigen (HBsAg) and iv) absence of hepatotoxicity events two months prior to the baseline visit. Clinical visits and blood tests were scheduled at the moment of initiation of the new ART, as well as at weeks 4, 12, 24, 36 and 48. The follow-up was stopped if the patient initiated treatment against HCV or at the moment of discontinuation of one or more antiretroviral drug due to any cause.

### Antiretroviral Drug Regimens

ART was indicated following the guidelines of the Spanish Group for the Study of AIDS (Grupo Español para el Estudio del SIDA, GESIDA), the Department of Health and Human Services of the USA (DHHS) or the European AIDS Clinical Society (EACS) at the time of prescription [21–23]. The final decision on the specific drug used was made according to the caring physician.

### Definition of Liver Toxicity

Grade 3 and 4 TE were defined according to the baseline alanine aminotransferase (ALT) and apartate aminotransferase (AST) values [24]. In patients with normal baseline transaminase levels, grade 3 TE were considered when elevations between 5 and 10 times above the upper level of normality (ULN) were observed at least one of the follow-up visits, while grade 4 TE were defined as ALT or AST values higher than 10 times of the ULN. In patients with elevated baseline transaminase levels, 3.5- to 5-fold elevations were considered grade 3 TE, and elevations higher than 5-fold were defined as grade 4 TE, respectively. Grade 4 TBE were defined as bilirubin levels exceeding 5 mg/dL.

### Statistical Analysis

The outcome variable of this study was the development of grade 3 or 4 TE during follow-up. Additionally, grade 4 TBE were analyzed as secondary outcome variable. Comparative analyses of TE and TBE were carried out for the different antiretroviral drugs that were initiated in this study, as well as for age, sex and other potential factors that could influence the development of grade 3 or 4 TE or grade 4 TBE. Continuous variables were expressed as median (IQR) and were compared using the Student’s *t*-test for normal distribution and the Mann-Whitney *U*-test otherwise. Categorical variables were expressed as number [percentage; 95% confidence interval (CI)] and were analyzed by means of the *χ*^2^-test or the Fisher’s test, when applicable. In order to compare the CD4 cell counts and the HIV viral loads at baseline and the end of follow-up, the Wilcoxon Signed Rank test and the McNemar test were applied, respectively. Finally, a logistic regression model was created with those factors that showed an association with the outcome variables in the univariate analysis with a p<0.2, as well as age and sex, in order to identify independent risk factors for the outcome variables. The adjusted odds ratios and the respective 95% CI were calculated. Statistical analysis was performed using the SPSS statistical software package release 22.0 (IBM, Chicago, IL, USA) and STATA 9.0 (StataCorp LP, College Station, TX, USA).

### Ethical Aspects

The study was designed and performed according to the Helsinki declaration and was approved by the Ethics Committee of the Valme University Hospital (Seville, Spain; approval reference number: 4/2011) and the Spanish Agency for Drugs and Sanitary Products (Agencia Española de Medicamentos y Productos Sanitarios, AEMPS). All patients gave their written informed consent before being included in the study.

## Results

### Study Population

A total of 192 patients were included in the analysis. The median (IQR) follow-up of the study population was 11.6 (5.6–12.9) months and the median (IQR) nadir CD4 cell count was 183 (67.8–274) cells/mL. Sixty-five (33.9%) individuals had received treatment against HCV infection without reaching sustained virologic response. Of these, 38 (58.5%) were non-responders, 8 (12.3%) had discontinued due to adverse events, 7 (10.8%) individuals had relapsed and 4 (6.2%) patients had developed a virologic breakthrough. The remaining 8 (12.3%) patients had dropped out voluntarily. Patient characteristics at the moment of starting a new ART combination are displayed in Table 1. Forty-one (21.4%) individuals started the new ART due to virologic failure, 55 (28.6%) patients due to adverse events to prior ART and 41 (21.4%) individuals had been ART-naïve. Simplification was the reason for switching in 29 (15.1%) individuals and in 20 (10.4%) patients changes were applied in order to avoid pharmacologic interactions with future therapy against HCV infection or other drugs. The remaining 6 (3.6%) changes were due to various reasons.

**Table 1:** Baseline characteristics of the study population (n = 192).

Characteristic	Value
Male sex, n (%)	169 (88)
Age, years*	46.4 (42.9–50.6)
Prior injection drug users, n (%)	150 (78.1)
Alcohol consumption ≥ 50 g/day, n (%)	30 (15.6)
CDC category C, n (%)	63 (32.8)
CD4 cell count, cells/mL*	393 (239–566)
Undetectable HIV viral load, n (%)	77 (40.1)
HCV-RNA (+)/HBsAg (-), n(%)	182 (94.8)
HCV-RNA (-)/HBsAg (+), n (%)	9 (4.7)
HCV-RNA (+)/HBsAg (+), n(%)	1 (0.5)
ALT*, IU/mL	50 (33–76.8)
AST*, IU/mL	46 (32.3–71.8)
Total bilirubin*, mg/dL	0.63 (0.44–0.9)
Liver stiffness*, kPa	8.5 (6.7–14.8)
Advanced fibrosis, n (%)^**§**^	84 (43.8)
Cirrhosis, n (%)^**§**^	55 (28.6)
Patients with hepatic decompensations prior to study, n (%)^#^	9 (4.7)

*Median (interquartile range)

^§^determined by transient elastometry: cut-off values were 9.5 kPa for advanced fibrosis and 14.6 kPa for cirrhosis

^#^number of events: ascites: 5, hepatic encephalopathy: 4, variceal hemorrhage: 2, jaundice: 1, hepatocarcinoma: 2.

### Newly Introduced Drugs and Resulting ART

A total of 342 drug initiations were registered. Among the newly introduced regimens, 77 (40.1%) involved a NRTI, 89 (46.4%) a PI/r and 77 (40.1%) a NNRTI, respectively. The detailed proportions of the drugs within these groups that were subject to change are displayed in Table 2. The majority [157 patients (81.8%)] received triple therapy. Eighteen subjects (9.4%) received dual therapy, 11 (5.7%) individuals received monotherapy, and a regimen consisting of four drugs was administered in 6 (3.1%) subjects.

**Table 2:** Newly introduced antiretroviral therapy (ART).

Antiretroviral drug	n (%)
Nucleoside analogue reverse transcriptase inhibitors (NRTI)	
Tenofovir/emtricitabine	49 (25.5)
Abacavir/lamivudine	14 (7.3)
Other NRTI combinations	14 (7.3)
NRTI-sparing	115 (59.9)
Ritonavir-boosted protease inhibitors	
Lopinavir/ritonavir	11 (5.7)
Atazanavir/ritonavir	25 (13)
Darunavir/ritonavir	53 (27.6)
Non-nucleoside analogue reverse transcriptase inhibitors	
Efavirenz	18 (9.4)
Nevirapine	7 (3.6)
Etravirine	16 (8.3)
Rilpivirine	36 (18.8)
Integrase inhibitors	
Raltegravir	24 (12.5)
Entry inhibitors	
Maraviroc	9 (4.7)

### Follow-Up

**Fig 1:**
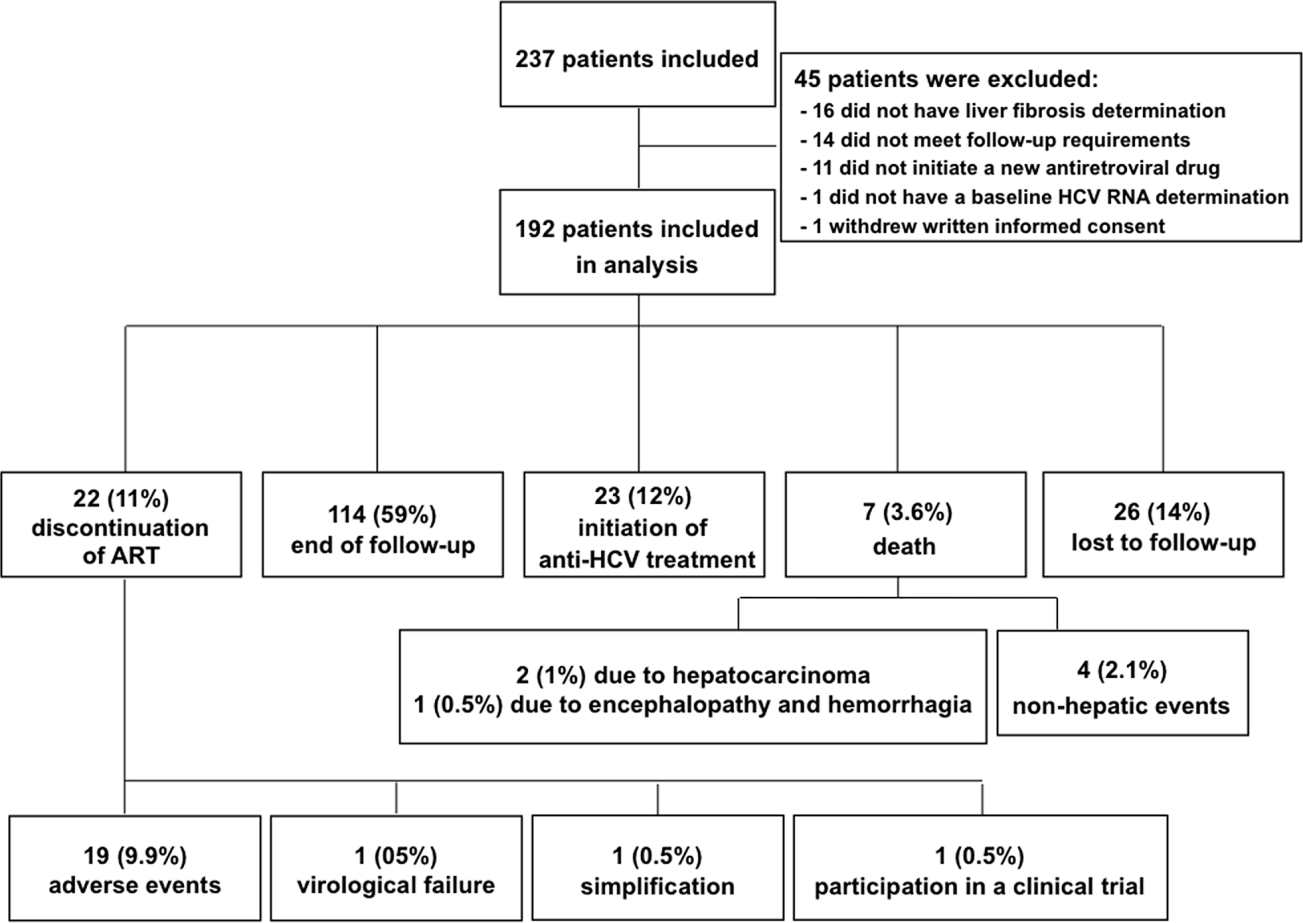
Flow chart for patient disposition and treatment outcome.

One-hundred and fourteen (59.4%) patients remained with the new ART until the end of the study follow-up of 48 weeks. The detailed reasons for ART discontinuation or premature stop of follow-up are depicted in Fig 1. The proportion of patients with undetectable HIV RNA was 40% (77/192 patients) at baseline and 81.6% (93/114 individuals) among those who reached the end of follow-up (p<0.001). CD4 levels (IQR) changed from 393 (239–566) cells/mL at baseline to 472 (298–712) cells/mL at the end of follow-up (p = 0.006).

#### Liver safety

A total of 10 (5.21%; 95%CI: 2.53%-9.37%) individuals developed grade 3 TE: 9 (4.9%) of the HIV/HCV-coinfected patients versus 1 (11.1%) of the HIV/HBV-coinfected individuals. No case of grade 4 TE was observed. Regimens and coinfection status of these patients are listed in Table 3. TBE was observed in 8 (4.17%; 95%CI: 1.82%-8.04%) patients, all of whom were HCV-coinfected and none showed HBV infection. Five (62.5%) individuals who presented TBE received ART based on ritonavir-boosted atazanavir and one respective patient received ritonavir-boosted darunavir, raltegravir or rilpivirine. No patient discontinued therapy due to hepatotoxic events. Liver decompensations during follow-up were developed by 8 (4.2%) patients, none of whom presented grade 3 or 4 TE or grade 4 TBE. In the multivariate analysis (Table 4), no factor was independently associated with grade 3 or 4 TE. The use of ritonavir-boosted atazanavir was the only independent predictor for grade 4 TBE.

**Table 3:** Patient characteristics of those who suffered grade 3 or 4 transaminase elevations (TE).

No.	Previous regimen	New	Months	Grade	Anti-	HBsAg
		regimen	to TE	of TE	HCV	
1	TDF/FTC/ETV	LPV/r	3	3	pos	neg
2	TDF/3TC/ATV/r	DRV/r/RAL	12	3	pos	neg
3	naïve	TDF/FTC/ATV/r	1	3	pos	neg
4	TDF/FTC/LPV/r	DRV/r	12	3	pos	neg
5	naïve	TDF/FTC/ATV/r	3	3	pos	neg
6	TDF/FTC/EFV	ABV/FTC/EFV	9	3	pos	neg
7	TDF/FTC/FPV/r	TDF/FTC/DRV/r	1	3	pos	neg
8	DRV/r/ETV/RAL	DRV/r/ETV/MVC	3	3	pos	neg
9	TDF/FTC/EFV	TDF/FTC/NVP	3	3	pos	neg
10	naïve	TDF/FTC/DRV/r	1	3	neg	pos

TDF: tenofovir; FTC: emtricitabine; ETV: etravirine; LPV/r: ritonavir-boosted lopinavir; 3TC: lamivudine; ATV/r: ritonavir-boosted atazanavir; DRV/r: ritonavir-boosted darunavir; RAL: raltegravir; EFV: efavirenz; ABV: abacavir; FPV/r: ritonavir-boosted fosamprenavir; MVC: maraviroc; NVP: nevirapine.

**Table 4:** Univariate and multivariate analysis of factors associated with grade 3 or 4 transaminase elevations (TE) and grade 4 total bilirubin elevations (TBE).

	n	Grade 3 or 4	*p*	AOR	*p*	Grade 4	*p*	AOR	*p*
		TE,	uni-	(95% CI)	multi-	TBE,	uni-	(95% CI)	multi-
		n (%)	variate		variate	n (%)	variate		variate
Age									
<46 years	96	7 (7.3)	0.194	1.027	0.57	5 (5.2)	0.360	1.052	0.438
≥46 years	96	3 (3.1)		(0.936–1.127)		3 (3.1)		(0.926–1.193)	
Sex									
Male	169	9 (5.3)	0.658	1.073	0.949	7 (4.1)	0.647	1.297	0.826
Female	23	1 (4.3)		(0.124–9.303)		1 (4.3)		(0.127–13.225)	
Alcohol intake									
<50 g/day	162	7 (4.3)	0.192	0.387	0.202	8 (4.9)	0.250		
≥50 g/day	30	3 (10)		(0.09–1.66)		0			
ALT levels									
≥40 IU/mL	121	7 (5.8)	0.458			6 (5)	0.378		
<40 IU/mL	71	3 (4.2)				2 (2.8)			
CDC category C									
Yes	63	3 (4.8)	0.574			2 (3.2)	0.479		
No	129	7 (5.4)				6 (4.7)			
Undetectable HIV RNA									
Yes	77	3 (3.9)	0.376			6 (7.8)	0.047	0.229	0.103
No	115	7 (6.1)				2 (1.7)		(0.039–1.344)	
CD4 cell count									
<350 cells/mL	81	5 (6.2)	0.421			3 (3.7)	0.753		
≥350 cells/mL	111	5 (4.5)				5 (4.5)			
Baseline cirrhosis									
Yes	55	2 (3.6)	0.415			3 (5.5)	0.414		
No	137	8 (5.8)				5 (3.6)			
Start of a NRTI									
Yes	77	3 (3.9)	0.503			5 (4.3)	0.593		
No	115	3 (4.5)				3 (3.9)			
Start of a PI/r									
Yes	89	7 (7.9)	0.124	1.319	0.733	4 (4.5)	1		
No	103	3 (2.9)		(0.269–6.475)		4 (3.9)			
Use of ATV/r									
Yes	29	2 (6.9)	0.649			5 (17.2)	0.002	7.327	0.018
No	163	8 (4.9)				3 (1.8)		(1.417–37.89)	
Start of a NNRTI									
Yes	77	1 (1.3)	0.052	0.183	0.748	1 (1.3)	0.147	0.397	0.43
No	115	9 (7.8)		(0.017–1.925)		7 (6.1)		(0.04–3.932)	
Start of RAL									
Yes	24	1 (4.2)	0.683			1 (4.2)	1		
No	168	9 (5.4)				7 (4.2)			
Start of a MVC									
Yes	9	1 (11.1)	0.389			0	0.676		
No	183	9 (4.9)				8 (4.4)			

AOR: adjusted odds ratio; CI: confidence interval; NRTI: nucleot(s)ide reverse transcriptase inhibitor; PI/r: ritonavir-boosted protease inhibitor; ATV/r: ritonavir-boosted atazanavir; NNRTI: non-nucleot(s)ide reverse transcriptase inhibitor; RAL: raltegravir; MVC: maravir

## Discussion

To our knowledge, this is the first study to evaluate the hepatic safety of current ART administered in clinical practice within a single prospective cohort consisting of a large sample of patients with chronic viral hepatitis. The frequency of grade 3 or 4 TE, as well as grade 4 TBE, associated with frequently used antiretroviral drug combinations in clinical practice is low in this setting.

To date, little information on hepatic safety of multiple ART regimens under current real-life conditions is available [4,5,20]. Our group reported in 2011 a frequency of 7.6% of grade 3 to 4 TE among HIV/HCV-coinfected patients, with no impact of the ART applied [20]. However, only efavirenz and PI/r were considered in this study, and the number of patients who received ritonavir-boosted atazanavir and ritonavir-boosted darunavir was low. Thus, that cohort does not reflect the current situation and indeed, in the present study, ritonavir-boosted atazanavir and ritonavir-boosted darunavir represent the most frequently prescribed PI/r, which is in accordance with international guidelines applied during the inclusion period, while potentially hepatotoxic drugs like saquinavir [25] and fosamprenavir [26] were not among the applied regimens. Furthermore, a considerable proportion of the patients received newer NNRTI, as well as maraviroc and raltegravir. It is to note that the frequencies of grade 3 or 4 TE and grade 4 TBE are low in this setting and was to be contributed to a specific drug. Furthermore, the frequencies of these hepatotoxicity events have decreased as compared to what was observed in a similar population from 2007 to 2009, analysed in the same centers and with a comparable study design [20], although they are somewhat higher as described in a different cohort [6]. Currently used ART can generally be considered to be well tolerated. In fact, no grade 4 TE was observed, and no discontinuation due to TE or TBE was reported. These are important findings, since they imply that the frequency of hepatotoxic events in HIV/HBV- and/or HCV-coinfected has decreased with the newer regimens as compared to what was reported at the beginning of the era of highly active ART [27–30]. Finally, the outcome of TE is good and discontinuations are of lesser concern, which results in a lower exploitation of treatment options and an augmentation of life quality for the patient.

In the history of safety studies involving antiretroviral drugs, the question of whether the presence of advanced fibrosis has an impact on the prevalence of ART-induced hepatotoxic events has risen especially regarding the hepatitis virus coinfected population [31,32]. However, various cohort studies published afterwards failed to confirm a relationship between the fibrosis stage and liver enzyme elevations in patients treated with various regimens [14–16,19,20] and an impact of advanced liver damage on grade 3 or 4 TE is therefore unlikely. Nevertheless, especially cirrhotic patients may have plasma levels of antiretrovirals that are above the safety concentration and HCV infection itself may have an impact on plasma levels of ART components such as ritonavir-boosted atazanavir, efavirenz and etravirine [33–35]. In the present study, a considerably high number of patients were administered the antiretroviral drugs susceptible to suffer plasma level elevations due to advanced liver disease. It is to point out that no association between cirrhosis at baseline and grade 3 or 4 TE or grade 4 TBE was observed.

A considerable number of patients started a new ART regimen with the prospect of possible initiation of treatment against HCV infection. This is not surprising since the study was conducted at the beginning of the era of direct-acting antivirals (DAA) against hepatitis C. In this context, in 2012, the protease inhibitors telaprevir and boceprevir were the first DAA approved for treatment against chronic hepatitis C in HIV/HCV-coinfected patients in Europe [23]. Unfortunately, drug-drug interactions resulted in contraindications for the concomitant use of these drugs with ritonavir-boosted lopinavir or ritonavir-boosted darunavir and required dose adjustments of telaprevir if ART included efavirenz [36,37]. Although with the next-generation DAA drug-drug interactions may be of lesser concern, this issue remains an important consideration, especially regarding HCV PI and will likely be reflected in the composition of future ART regimens [23]. In the present study, the considerably high proportion of patients who initiated ritonavir-boosted atazanavir account for this finding. We point out that the use of ritonavir-boosted atazanavir was independently associated with grade 4 TBE, which stands in accordance with former studies [13,38,39]. However, atazanavir-induced hyperbilirubinemia did not lead to treatment interruptions and was not associated with the clinical outcome of ART in the short and the long term [38,39]. Although bilirubin levels should be monitored in this setting, a clinical impact of this phenomenon is unlikely.

Sustained virological response to therapy against HCV infection leads to a marked decrease in the risk of drug-related liver toxicity in HIV/HCV-coinfected patients [40]. This fact prompted the recommendation of treating hepatitis C before HIV-infection in subjects with high CD4 cell count [21–23]. However, as shown here, the risk of severe liver toxicity with the currently used drugs is very low. In addition, the results of the START Study [41] support immediate ART after HIV infection diagnosis. Because of these reasons, and given that the interactions of antiretroviral drugs and direct-acting antivirals are manageable, in our opinion, deferring ART until having treated chronic hepatitis C infection is no longer justified in HIV/HCV-coinfected subjects.

This study has limitations. Due to the high variety of possible ART regimens, some drugs were applied to a lesser extent. Still, the antiretroviral drugs approved at the time of the recruitment of patients were administered in an adequate number and the composition of the regimens studied herein well reflect the situation of ART during the inclusion period. Finally, most recently approved drugs like dolutegravir and elvitegravir/cobicistat were not available during patient recruitment and should be considered in future study designs.

In conclusion, currently applied ART regimens can be considered safe in HIV-infected patients with viral hepatitis and advanced liver damage did not impact on the development of severe laboratory abnormalities analyzed herein. Although the frequencies are similar to what has been reported previously, the severity of hepatotoxic side effects have decreased throughout the last years and are unlikely to lead to treatment discontinuations.
